# Know-how of holding a Bioinformatics competition: Structure, model, overview, and perspectives

**DOI:** 10.1371/journal.pcbi.1011679

**Published:** 2023-12-21

**Authors:** Elvira C. A. Horácio, Lucas M. de Carvalho, Gustavo G. Pereira, Mayla C. Abrahim, Mônica P. Coelho, Deivid A. De Jesus, Glen J. Y. García, Raquel C. de Melo-Minardi, Sheila T. Nagamatsu

**Affiliations:** 1 Postgraduate Program Genetics, Institute of Biological Sciences, Federal University of Minas Gerais, Minas Gerais, Brazil; 2 Rene Rachou Institute, Oswaldo Cruz Foundation, Minas Gerais, Brazil; 3 Center for Computing in Engineering and Sciences, State University of Campinas, São Paulo, Brazil; 4 Computer Scientist, Franciscan University, Rio Grande do Sul, Brazil; 5 Laboratório de Tecnologia Imunológica, Instituto de Tecnologia em Imunobiológicos, Vice-Diretoria de Desenvolvimento Tecnológico, Bio-Manguinhos, Fundação Oswaldo Cruz (FIOCRUZ), Rio de Janeiro, Brazil; 6 Division of Clinical Immunology and Allergy, Medical Research Laboratory, School of Medicine - University of São Paulo, São Paulo, Brazil; 7 Graduate Program in Genetics. Institute of Biology. Federal University of Rio de Janeiro, Rio de Janeiro, Brazil; 8 Bioinformatics Graduate Program, Institute of Biological Sciences, Federal University of Minas Gerais, Minas Gerais, Brazil; 9 Computer Science Department, Institute of Exact Sciences, Federal University of Minas Gerais, Minas Gerais, Brazil; 10 Yale University, School of Medicine, New Haven, Connecticut, United States of America; SIB Swiss Institute of Bioinformatics, SWITZERLAND

## Abstract

The article presents a framework for a Bioinformatics competition that focuses on 4 key aspects: structure, model, overview, and perspectives. Structure represents the organizational framework employed to coordinate the main tasks involved in the competition. Model showcases the competition design, which encompasses 3 phases. Overview presents our case study, the League of Brazilian Bioinformatics (LBB) 2nd Edition. Finally, the section on perspectives provides a brief discussion of the LBB 2nd Edition, along with insights and feedback from participants. LBB is a biannual team competition launched in 2019 to promote the ongoing training of human resources in Bioinformatics and Computational Biology in Brazil. LBB aims to stimulate ongoing training in Bioinformatics by encouraging participation in competitions, promoting the organization of future Bioinformatics competitions, and fostering the integration of the Bioinformatics and Computational Biology community in the country, as well as collaboration among participants. The LBB 2nd Edition was launched in 2021 and featured 251 competitors forming 91 teams. Knowledge competitions promote learning, collaboration, and innovation, which are crucial for advancing scientific knowledge and solving real-world problems. In summary, this article serves as a valuable resource for individuals and organizations interested in developing knowledge competitions, offering a model based on our experience with LBB to benefit all levels of Bioinformatics trainees.

## Author summary

There are various methods of learning, and one such approach involves a commonly used in the computing field, fostering competition, where communication, teamwork, group learning, and problem-solving play crucial roles. Similarly, in bioinformatics and computational biology, certain institutions or platforms promote learning through challenges. However, national-level competitions in these domains are relatively scarce. Here, we would like to present a framework for a Bioinformatics competition by showing structure, model, overview, and perspectives, using the League of Brazilian Bioinformatics (LBB) 2nd Edition as case study. LBB is the first bioinformatics competition in Latin America and aims to encompass the diverse multidisciplinary aspects of Bioinformatics and promote networking. In LBB, individuals not only have the opportunity to learn but also get to know one another. Further, to stimulate undergraduate training, we promoted teams with a mixture of different academic backgrounds. This approach promotes integration among participants and facilitates the exchange of information. To ensure that all participants have the opportunity to connect and form teams regardless of their background, we have created the MATCH, a script that allows individuals to be randomly paired with others, enabling them to get to know each other and create teams effectively.

This approach fosters collaboration and creates a conducive environment for learning and problem-solving.

## 1. Introduction

In recent years, there has been a growing literature recognizing competitive elements as an alternative of pedagogical approach across diverse fields [1–6]. Regardless of the field, the introduction of competition can foster engagement, collaboration, and the development of essential skills, such as cultivation of problem-solving, real-world relevance, critical thinking and creativity, interdisciplinary skill development, time management, collaboration and teamwork [7]. This phenomenon has gathered attention due to its potential to enhance learning outcomes by simulating real-world challenges and promoting active participation. A study has shown that the application of a hackathon called “Markathon” can stimulate learning in marketing [8]. The study evaluated 92 students and found that participating in the Markathon has improved their ability to identify and solve problems, apply innovation skills, think critically, among other skills [8]. The mean of the responses was higher than 4, where 5 indicates strong agreement [8]. Another study, which introduced a novel educational model designed to teach essential skills for medical innovation to students from diverse backgrounds (*n* = 161), demonstrated that participants acquired substantial knowledge in all 10 categories assessed in the study. Notably, there was a 166.7% increase in understanding the healthcare regulatory landscape and a 38.3% increase in hardware prototyping [9]. While we lack studies in the context of Bioinformatics education, the integration of competitive aspects holds particular promise as it can be beneficial for improving learning. This arises from its complexity, which requires multidisciplinary knowledge and programming skills. This underscores the significance of practice over mere knowledge, enabling the accurate application of knowledge to solve problems, in contrast to the experiences in knowledge-based fields. Notably, various field-specific competitions, including the Critical Assessment of Protein Structure Prediction (CASP) [10] and the Wikipedia competitions [11], have demonstrated the educational advantages of competitive platforms besides creating innovative tools as the AI system Alphafold developed during CASP14 by DeepMind [12]. Moreover, recent instances such as the Bioinformatics Contest [13] further underscore the potential of competition to invigorate Bioinformatics education in problem-solving challenges.

Taking this into consideration, the League of Brazilian Bioinformatics (LBB) was launched in 2019 as a biannual team challenge. It was established in Brazil to advance the continuous development of human resources in Bioinformatics and Computational Biology. LBB strives to invigorate continuous skill enhancement in Bioinformatics by inspiring engagement in competitive events, facilitating the planning of future Bioinformatics competitions, and nurturing unity and collaboration within the nation’s Bioinformatics and Computational Biology community.

In this article, we introduce a comprehensive framework for a Bioinformatics competition that places emphasis on 4 pivotal elements: structure, model, overview, and perspectives. Firstly, “Structure” encapsulates the meticulously designed organizational framework that plays a crucial role in orchestrating the various tasks integral to the competition’s success. Secondly, “Model” delves into the intricacies of competition design, offering insight into the 3 distinct phases that shape the competition’s format and execution. Moving forward, “Overview” directs our attention to a captivating case study—the LBB 2nd Edition—providing a comprehensive exploration of this noteworthy event. Lastly, the section devoted to “Perspectives” offers a concise yet enlightening discussion surrounding the LBB 2nd Edition, replete with valuable insights and candid feedback garnered from the enthusiastic participants.

## 2. Results and discussion

Brazil is a pioneer of Bioinformatics in Latin America and has a wide range of talents that are potential recipients of training, each with different needs in terms of what skills and/or knowledge to develop. With that in mind, in order to promote their training by applying Bioinformatics and Computational Biology tools to solve multidisciplinary challenges that they may face in the real world, we launched the LBB [14] in 2019, a biannual competition conceived by the Regional Student Group Brazil (RSG-Brazil) [15], a student organization affiliated with the International Society for Computational Biology—Student Council (ISCB-SC) [16], in collaboration with the Brazilian Association of Bioinformatics and Computational Biology (AB3C) [17].

LBB has as main objectives: (1) stimulate the continued training of human resources in Bioinformatics through participation in competitions; (2) stimulate and promote the organization of future Bioinformatics competitions both nationally and internationally; (3) promote the integration of the Bioinformatics and Computational Biology community in the country and the collaboration between the participants. Our objectives are in line with the ISCB’s bioinformatics “core competencies” that aspiring professionals in this field should acquire [7]. By referencing these competencies, the participation into LBB competition can improve general biology, depth knowledge in at least one area of study, scientific discovery process, Bioinformatics tools usage, ability in several programs in scientific problems, command line and script development, effective team working and communication, and engaging in a continuing professional development in Bioinformatics.

In the LBB 2nd Edition (2021), we made improvements in the organizational structure and went further with the competition by implementing workshops, lectures, and networking sessions. Specifically, we arranged a comprehensive lecture series for the broader audience, alongside numerous smaller events tailored for the participants. We aimed to increase the engagement of competitors, improve their knowledge of Bioinformatics, and stimulate soft skills. Furthermore, given the significance of Bioinformatics in tackling major issues in the life sciences, replicating similar competitions in different regions and countries could boost the expansion and progress of the global bioinformatics community engaging more students and young researchers. This work offers a guide on creating a bioinformatics competition and shares our experience to encourage knowledge competition replication for all bioinformatics trainee levels.

### 2.1 Structure: Organizational framework

**Fig 1:**
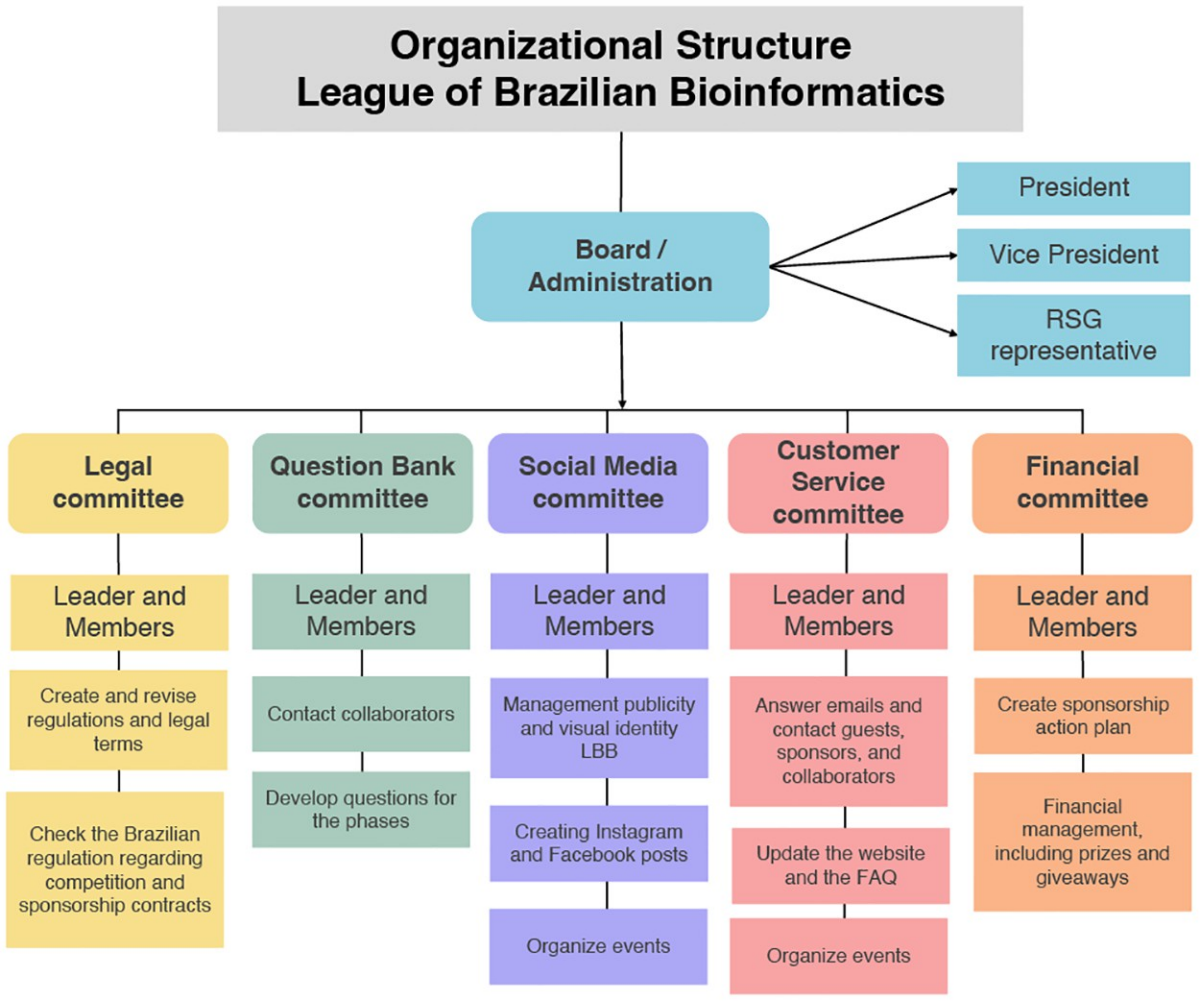
LBB organizational structure. The organization comprises administrative and 5 committees, which include Customer Service, Question Bank, Financial, Legal, and Social Media. Additional information can be located in Box 1.

Ensuring the success of a Bioinformatics competition largely depends on having a well-defined structure and clear processes. The organizational structure was divided into 5 committees, namely, the Question Bank, Social Media, Customer Service, Legal, and Financial (Box 1; Fig 1). Additionally, the main board consisted of a president, a vice president, and a representative of RSG-Brazil, who acted as an intermediary to coordinate any needs that might arise with the organization. Our structure was designed in a way we would be able to manage the competition and account for possible conflict of interest.

**Box 1. Structure the organization.**

LBB is structured based on roles and committees, group of members responsible for managing a specific role. The strategic direction of LBB is led by the president, who is also responsible for setting the goals for the edition and representing the organization to external stakeholders and the general public. The vice president provides support to the president and assumes leadership in the case of a leave of absence. The RSG-Brazil representative acted as an intermediary between the LBB board and the RSG-Brazil board, in order to coordinate any needs that may arise between the organizations. All members of the organization participated in at least one of the 5 working committees, led by a leader who set the goals and coordinated the deliverables. The Question Bank committee was in charge of managing the tasks involved in the 3 phases of the competition. For the first stage, they developed 60 original questions (20 in Computer Science, 20 in Biology, and 20 in Bioinformatics), ensuring to cover a wide variety of topics from each of the 3 main areas. Additionally, this committee was responsible for contacting potential collaborator professors, managing the deliverables, developing (when required) challenges for the second stage, and preparing the third phase of the competition. The Legal and Financial Committee was responsible for drafting the competition announcement, and the general rules, including the legal framework that established the permits and obligations that each participant had to comply with. The legal team was responsible for developing the responsibility term, image use authorization, and the code of conduct.

In addition, they were responsible for obtaining support, and the assistance required to execute the event. They were also in charge of seeking sponsors to cover administrative expenses and award prizes to the finalist teams. These commissions can be joined in a technical way to facilitate the referral; however, it is necessary to verify the legislation in which the competition is being held. The Social Media Committee was responsible for developing communication and digital marketing strategies for the competition, creating and publishing content on Facebook, Instagram, and the website. The content included Bioinformatics knowledge, information about the competition cycle, and additional events, such as seminars, conference cycles, networking, and the Cicada contest. Additionally, the committee interacted with the general public to answer questions and comments on social media. Generating a broader accessibility to a broad audience (e.g., undergraduate, graduate, and professional), in addition to accounting for regional accessibility. The Customer Service Committee was responsible for providing high-quality formal assistance to participating teams and the general public (individuals not registered in the competition) such as technical issues about registration, exams, and internal and external events. They managed the website by being responsible for updates and FAQ. And they have coordinated and developed conference cycles and networking events, in which they send out invitations related to the competitions and events. They worked closely with the Social Media Committee, complementing each other to ensure effective interaction with the public and the success of the competition. Each committee has the autonomy to hold weekly, fortnightly, or monthly meetings according to their demand. Monthly general meetings are organized to comprise the whole organizational committee, where each work commission presents its progress. During these meetings, any eventuality was resolved democratically and always with the agreement of the majority of the LBB members (S1 Text).

### 2.2 Model: Competition design

#### 2.2.1 Application guidelines

Application guidelines were announced on the first day of registration. The document contained comprehensive information covering general provisions, goals, participation requirements, subscription details, roles of the Organizing Committee and LBB Coordinators, structure of evidence and correction criteria, evaluation test locations, awards, and final considerations (S1 Text). In essence, participants from a wide range of educational backgrounds were permitted to register as team members, including high school, undergraduate, graduate, and professional students. The following criteria were to be met: (1) be at least 18 years old at the time of registration; (2) have no more than 2 years since obtaining their PhD degree; (3) have less than 4 years of experience in a bioinformatics-related field; (4) be proficient in understanding Portuguese; and (5) be part of a team consisting of 2 or 3 members, with at least 1 member affiliated with a Brazilian institute. Additionally, the team should not include more than 1 member with a PhD degree. The team was required to select a name, which was used by the Question Bank Committee to uniquely identify teams and prevent conflicts of interest during evaluation. Furthermore, participants were obligated to adhere to a code of conduct that prohibited discriminatory behavior and unethical conduct throughout the competition. Internally, all information was treated confidentially, and the Organizing Committee required members to sign a confidentiality agreement. This agreement allowed them to share only accessible information with the 2 organizers’ groups (RSG-Brazil and AB3C) and the general public. This measure was implemented to prevent any participant from gaining an unfair advantage through foreknowledge.

#### 2.2.2 Phases and evaluation tests

**Fig 2:**
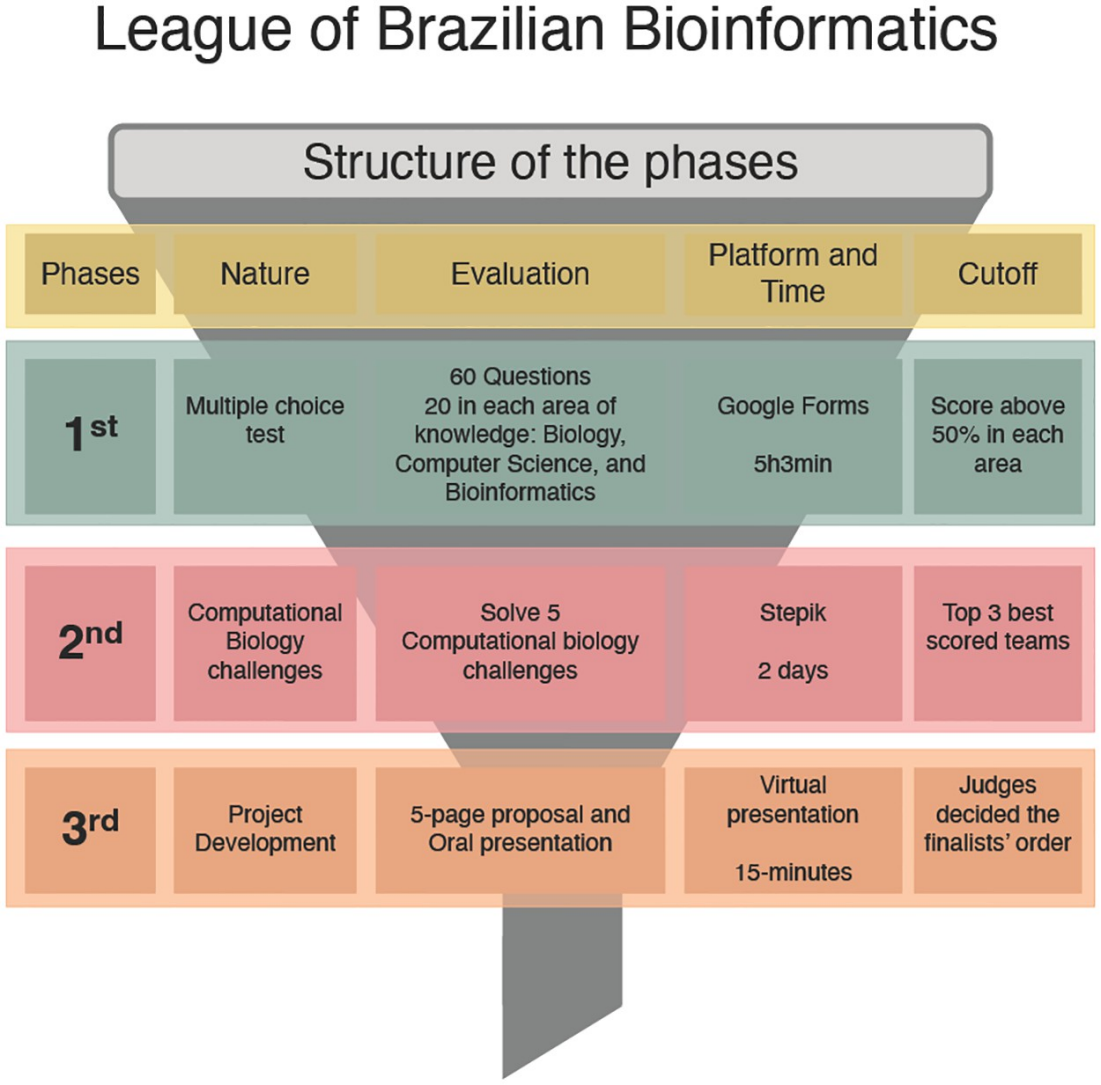
Model of the LBB. An overview of each phase’s execution and the evaluation criteria for cutoff and selection. The figure also includes the platforms used to develop the competition and the time allowed for each phase. Additional information is shown in S1 Text and Box 2.

The LBB followed a 3-phase evaluation (Box 2; Fig 2) that remained mostly consistent over the years. In the first phase, participants were given a set of 60 multiple-choice questions, divided equally among the 3 areas of Biology, Computer Science, and Bioinformatics. In the 2nd Edition, differently from the 1st Edition, the participants had to score at least 50% in each area to reach the second phase. The second phase involved 5 computational biology problems that participants had to solve, and the 3 groups with the best performance moved on to the final phase. In the final phase, participants were required to develop a bioinformatics-based project, which was judged based on its scientific quality, technical proficiency, and creativity.

**Box 2. Phases.**

The competition was divided into 3 phases held virtually. The first phase aimed to evaluate the diversified knowledge of the teams in the 3 main areas addressed in Bioinformatics and Computational Biology, Computer Science, Biology, and Bioinformatics. The second phase aimed to evaluate the teams’ ability to work together and solve biological problems using Bioinformatics. While the third phase aimed to evaluate research skills, including creativity, communication, and logical thinking.

- First phase: Teams should solve 60 multiple-choice questions in 5 hours and 3 minutes in allusion to the transcription direction 5’ to 3’. These questions were divided into 20 Biology, 20 Computer Science, and 20 Bioinformatics. The teams were required to get at least 50% of the questions right in each area of knowledge to qualify for the second phase. We assumed an exception to this rule in case fewer than 30 teams met this requirement, where the best-positioned teams would also be classified until completing the minimum number required. After the exam, teams had the opportunity to present claims if they found errors or ambiguities in any question, and the commission had the power to cancel such questions. To determine the ranking, the completion time of the evaluation test was taken into account as a tiebreaker criterion.
- Second phase: The qualified teams faced a series of Computational Biology challenges designed by professors from universities and research institutes specialized in Bioinformatics and/or by the organizational committee. If necessary, the Question Bank Committee adapted these challenges to adjust their complexity. The challenges were presented on the Stepik platform, which allowed the automatic correction of answers. The use of this platform also allowed the teams to review and correct their answers anytime until completing the deadline. The organizing committee promptly resolved any claims made by the teams in the first hours. The top 3 teams qualified for the next phase, and in case of a tie, the resolution time was used as a tiebreaker criterion, as well as the number of correct answers in the first phase.
- Third phase: It consisted of the presentation of a project designed by the Question Bank Committee. The committee selected a theme to be addressed, and the participants should develop a 3-year project in any Bioinformatics area, suggesting collecting their own data or using a public database. They should consider a budget of R$500,000.00. The 2nd Edition theme chose Plant Biodiversity as the main theme. The judge board was composed of 4 research professors invited to evaluate the teams. The teams were requested to write a 5-page project and give a 15-minute presentation with 40 minutes of questions. The judge board has the autonomy to decide the final rank.

The replication of the competition structure in both editions allowed for a consistent and fair assessment of participants’ skills and knowledge in the 3 areas. The multiple-choice questions in the first phase were designed to test a broad range of knowledge and skills, while the Computational Biology problems in the second phase provided a more in-depth assessment of participants’ ability to apply their knowledge to real-world problems. Moreover, group-based competitions focused on problem-solving are a promising approach for gathering valuable insights into how participants collaborate in teams and tackle complex, open-ended challenges [18–21]. The competition can yield rich data on team dynamics and problem-solving strategies employed by the participants.

Finally, the bioinformatics-based project in the third phase encouraged participants to think creatively and develop innovative solutions to challenges in the field. Overall, the 3-phase structure of the competition provided a rigorous and comprehensive evaluation of participants’ abilities and served as an effective platform for promoting talent and innovation in the field of Bioinformatics.

#### 2.2.3 Partner search, webinars, events, and networking

As a team competition, we encourage individuals to also participate by helping them to find a team. To address this need, we implemented different strategies. The main method was a semiautomatic MATCH platform to create a team, named LBB MATCH [22], in Python. When registering for LBB MATCH, each candidate fills out an electronic form describing their academic profile and the desired profile to compose their team. The script (S2 Text) would then select individuals from different undergrad areas and address the main rules of the competition, promoting the networking of people who would hardly work together.

Further, considering that most of the LBB participants were trainees, we endeavor to contribute to increasing their Bioinformatics knowledge and networking skills. For this, we hold webinars covering different topics (YouTube channel RSG-BRAZIL) [23], promoted lectures to complement their training, and networking sessions to introduce participants to professionals and to real problems and solutions within the field of Bioinformatics and Computational Biology. We make an effort to show a variety of areas and careers in the Bioinformatics field to the participants. However, we decided to take advantage of our position to open some of the seminars to the general public, covering broad areas and including underresearching topics. We also promoted the attendance of underrepresented groups. In this sense, we organized the LBB Cycle of Lectures, with introductory and advanced lectures covering themes like epigenetics, docking, and noncoding RNAs. In addition, we included 1 day to promote Bioinformatics and Computational Biology research in Brazilian institutions. The Cycle of Lectures had more than 200 online participants and reached more than 500 views per day by December of 2023. Further, to promote diversity, we quantitatively distributed the events to have a similar number of women and males talks in all LBB events. Further, we also did our best to diversify the institutions and Brazilian States in which the speakers worked, encouraging the inclusion of other groups in our event as well.

#### 2.2.4 Key considerations

The LBB competition is governed by a set of regulations that outline the rules and guidelines for participating in the event (S1 Text). These regulations cover various aspects of the competition, including eligibility, key dates, format and content of each phase, and evaluation criteria. In summary, the 2nd Edition’s regulations specify that LBB is open to teams (2 or 3 participants) who have any background and would like to test their knowledge and skills in a range of bioinformatics tasks and challenges. Overall, the regulations of the bioinformatics competition are aimed at ensuring a fair, transparent, and competitive environment, where participants can demonstrate their expertise and creativity, and where the broader bioinformatics community can learn and benefit from their contributions.

Taking into account the importance of reproducing the competition as a mechanism of promoting learning, we make an effort to register all the steps to build the LBB in a systematic way. In the Supporting information, we made available the regulations, organizational structure, codes, and methods to implement the LBB MATCH.

### 2.3 Overview: Data assessment and evaluation test of LBB 2nd Edition

**Fig 3:**
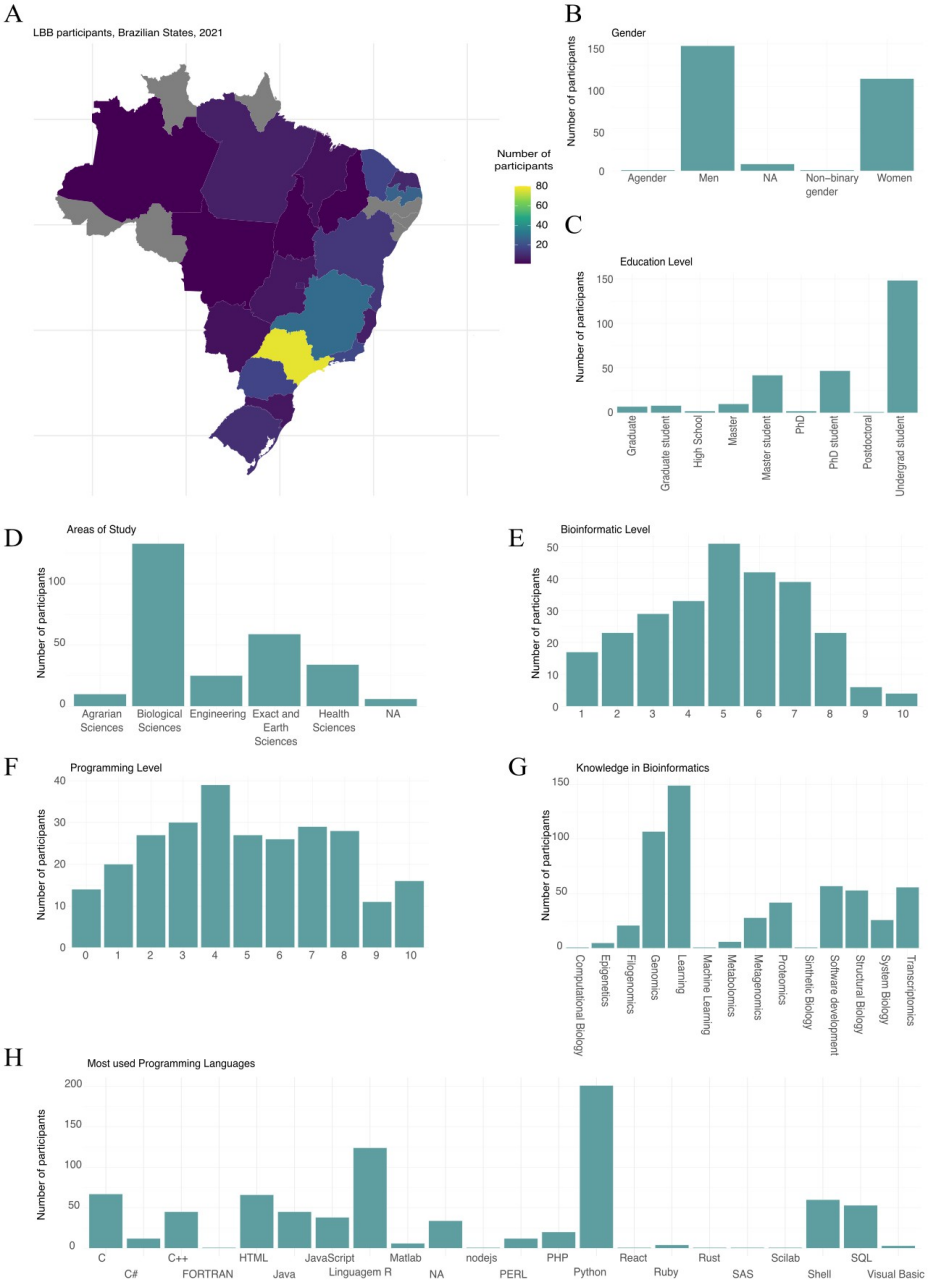
Demographics and knowledge information of participants of the LBB 2nd Edition. (**A**) Distribution of participants in the Brazilian states. The graph shows the count of participants from each Brazilian state who reported residing during the LBB 2nd Edition. (**B**) Self-reporter gender. We observed a higher percentage of men registered in the LBB 2nd Edition. (**C**) Self-reported education levels. The x-axis represents the highest level of education achieved or ongoing studies for each participant. (**D**) Fields of study during undergraduate studies. Each participant was asked to provide their area of study during their bachelor’s degree. (**E**) Bioinformatics level of knowledge. (**F**) Programming level. For (**E**) and (**F**), we have the distribution of self-reported knowledge for all participants in the context of bioinformatics and programming level, respectively. (**G**) Prior knowledge in Bioinformatics. We gathered information about participants’ prior knowledge in various bioinformatics areas, revealing genomics as the most commonly selected omics field. (**H**) Programming language used by the participants. Besides collecting information about programming level, we also identified the most used languages by the LBB 2nd Edition participants, observing a majority of Python users. All data used to prepare this figure are in Tables A-H in S1 Data.

During registration, self-reported demographic data and knowledge profiles were assessed. Here, we analyzed the composition of LBB 2nd Edition’s participants (Fig 3). Regarding the evaluation tests, we estimated the ability of the questions in the first phase to separate the teams of competitors between those with high and low ability to accurately address the question using item response theory (IRT) models [24]. We aimed to identify questions that proved to be effective during the first phase (Figs A and B in S3 Text). The IRT refers to a family of mathematical models that aim to explain the pattern of responses or performance and the relationship between latent traits (attribute or unobservable characteristic). For the second phase, we conducted a principal component analysis (PCA) with the score of each team. Here, we were also able to observe whether each question was able to divide the teams (Fig C in S3 Text).

#### 2.3.1 Demographic quantitative and participants profile

When comparing the demographic distribution of participants from the years 2019 (LBB 1st Edition) and 2021 (LBB 2nd Edition), there were notable differences in the expansion of the subscribers in the competition. Overall, the 1st Edition of LBB featured 168 competitors divided into 59 teams [25], while the 2nd Edition saw increased participation, with 251 competitors forming 91 teams. In both Editions, the majority of participants were from São Paulo and Minas Gerais states, with a significant number of participants from the Brazilian south region and a smaller number from other regions. These numbers are in agreement with the number of research groups in the Brazilian regions in the last census of the National Council for Scientific and Technological Development (CNPq) [26]. However, in the LBB 2nd Edition, we observed an increase in the number of states we were able to reach (Fig 3A), which surged from 18 to 23 states out of 27 Brazilian states plus the Federal District. This could be attributed to several factors, including our harder work on stimulating the LBB social media as well as the rise of virtual events due to the COVID-19 pandemic, which made it more accessible for people from different parts of the world to participate in programming events. Additionally, compared with LBB 1st Edition where the majority of our participants were self-declared males, there was a noticeable increase in the number of female participants in 2021 (31% in 2019 to 41.4% in 2021), suggesting that efforts to promote diversity and inclusion in the programming community may be starting to have an effect. In addition to our effort in increasing diversity in the LBB 2nd Edition, numerous initiatives have been undertaken to promote diversity in Brazil. Notably, LBB Foundation in 2019 was the result of collaborative efforts between RSG Brazil and individual students in the field. In that particular year (2019 to 2020), the RSG Brazil made a concerted effort to establish its first-ever women-only board and organize a student symposium centered around the theme of women in science. Simultaneously, in 2019, it was created the Women in Bioinformatics and Data Science Latin America community (WBDSLA) [27]. Later, additional initiatives were developed, such as the pyLadies [28], which play a pivotal role in fostering inclusivity and participation of minority groups such as women, blacks, indigenous people, and the LGBTQIA+ community. Decentralized and long-term actions have positive effects throughout the scientific community, in addition to being important actions in events that aim to address different professionals.

In terms of skills and programming ability, there was a marked improvement among participants from LBB 2nd Edition. We observed more people with basic programming skills than in the 1st Edition. However, due to limitations in our assessment and event promotion, we were unable to investigate the causal relationships behind the increase in their basic programming skills. At the same time, we observed many participants having higher expertise in multiple programming languages, suggesting the participation of computer scientists. This suggests that the bioinformatic community is evolving and attracting not only biologists but also highly skilled programmers. Additionally, there was a noticeable increase in the number of participants who were learning Bioinformatics. We also observed genomics as the most known field by the participants and an increase of participants with previous knowledge in proteomics compared with the 1st Edition. We also had few participants with knowledge of Epigenetics, not evaluated in the previous competition.

#### 2.3.2 Evaluation tests

To evaluate the first phase, we chose to apply the IRT based on the 2PL model to estimate the evaluation test and individual questions’ importance. The results from the IRT model provide valuable insights into the performance of the participants in the 3 areas of Biology, Computer Science, and Bioinformatics, as well as how their performance has changed over time. The IRT model proved to be effective in separating the groups based on their performance in the 3 areas, indicating that the questions were well designed and effectively measured the skills and knowledge of the participants (Fig A in S3 Text). The model was able to identify questions that were particularly important for distinguishing between high-performing and low-performing teams in each area, mainly in the Computer Science area, which could help inform areas of knowledge that should be implemented in future evaluation tests and educational programming (Fig B in S3 Text). Evaluating the LBB second phase results, we performed a PCA (Fig C in S3 Text), in which comparing with the results from the 1st Edition showed several similarities. Considering that both editions had the scores assigned to different teams ordered as the final team ranking, and the loadings representing the questions, we were able to compare the results. The distribution of the scores and loadings are correlated between the 2 analyses, indicating that the patterns of variation in the data have been maintained over time. Specifically, this analysis identifies questions that were essential for selecting the first-placed time in the competition. During the evaluation of the third phase, we decided to give the Judge the autonomy in deciding who was the best team, mirroring the format of Brazilian PhD and Masters’ thesis. Furthermore, we have asked the judges to take into account the presence of a well-defined scientific question, the appropriateness of methodologies employed to address that question, the thoroughness of exploration and interpretation of the obtained results, as well as the lucidity and ingenuity demonstrated in the project’s presentation. To avoid bias, we decided not to introduce the finalists’ backgrounds and selected professionals from the different areas of Bioinformatics and Computational Biology to the board. We also opted to not include anyone on the organizing committees, avoiding conflicts of interest.

### 2.4 Perspectives

Bioinformatics competitions have become increasingly important in promoting the development of the next generation of Bioinformatics talent worldwide. LBB is the first Bioinformatics competition in Latin America, so we allowed the registration of international participants who considered themselves able to read and interpret questions in Portuguese. Competitions provide a dynamic platform for students to showcase and enhance their skills and knowledge within the realm of bioinformatics. By engaging in these competitions, students not only immerse themselves in the excitement of solving intricate biological problems but also gain invaluable practical experience in utilizing a wide array of bioinformatics tools and techniques. These experiences directly align with the bioinformatics “core competencies” outlined by the ISCB [7], enabling students to acquire and refine essential proficiencies. Moreover, participation in these competitions offers exposure to cutting-edge research and industrial applications, further enriching students’ understanding of real-world bioinformatics challenges and opportunities. Additionally, they serve as a way to identify and reward the most talented and promising students and to encourage all participants to pursue careers in Bioinformatics and related fields. Feedback from participants, as well as insights from LBB organizers, are documented in Box 3. Future work should include self-evaluation of the knowledge acquired during the LBB to assess individual learning curves during a competition.

**Box 3. Organization’s opinion and Participant’s opinion.**

**Organization’s opinion**:

“Organizing LBB was both a tremendous challenge and a dream come true. The competition witnessed an exceptional level of competition, with participants showcasing their innovative problem-solving approaches and pushing the boundaries of bioinformatics. The process of orchestrating a national competition demanded vast dedication from our team and fostered a sense of trust among everyone involved. Personally, I feel a significant growth as a professional, and my leadership skills have flourished, leading to stronger relationships within the team.”

“Organizing the LBB was an enormous undertaking that presented us with numerous challenges. We encountered a range of obstacles and concerns, such as securing adequate funding, promoting active participation, and designing a competition that not only provided an enjoyable experience but also enriched participants’ backgrounds. The profound satisfaction we derived from witnessing the outcomes that ensued after investing our unwavering efforts was especially rewarding, particularly knowing that we played a part in advancing the field of bioinformatics in Brazil.”

“As an organizer, I emphasized the significance of people management and project management. I also acquired knowledge in bioinformatics, biology, and computing. Learning was crucial for developing diverse topics covered in webinars, events, and networking. Additionally, I recognized the impact of media management on different groups. Teamwork played a fundamental role in creating inclusive events that catered to various levels of study and encompassed a wide range of areas, even those unfamiliar to our audience.”

**Participant’s opinion**:

“The LBB in the year 2021 was sensational because, in addition to teams learning new things about biology, computer science, and bioinformatics, we also learned how to work as a team. Another fantastic aspect was the events, Bioinformatics lectures, and prizes that the teams won. I still use my LBB cup to this day.”

Compared to global programming competitions in Bioinformatics, such as the Bioinformatics Contest [13], the LBB 2nd Edition stands out due to its unique structure and emphasis on participant training. While both competitions involve programming phases centered around computational biology problems, LBB takes a different approach by organizing participants into groups. This group-based structure fosters interdisciplinarity and facilitates the exchange of knowledge among participants, setting it apart from individual-focused competitions. Furthermore, the LBB competition distinguishes itself by including a third phase that involves the presentation of a project following the research promotion agency standards in Brazil. This aspect implies that participants in LBB not only gain programming skills but also develop the capabilities of a researcher. This practical application of knowledge reinforces the real-world relevance of the competition and prepares participants for research-oriented endeavors. Lastly, LBB stands as the only competition that actively promotes enhanced interaction with participants through webinars and courses. By providing these additional educational resources, LBB fosters a richer learning experience and facilitates deeper engagement with the subject matter.

## 3. Materials and methods

All methods used in data analysis are described in S4 Text.

## Supporting information

S1 Text League of Brazilian Bioinformatics Regulation. The document presents the competition rules of the LBB 2nd Edition.
(DOCX)

S2 Text Script MATCH. The file contains the script used to create groups of participants following defined criteria.
(DOCX)

S3 Text Data analyses. This file includes the main analyses of the participants’ scores in the first and second phases.
(PDF)

S4 Text Methods. The document describes the methods used for the statistical analyses.
(DOCX)

S1 Data Data availability. The file contains the data to generate the figures presented in Fig 3.
(XLSX)

S2 Data Data availability. The file contains the data to generate the figures presented in Figs A and B in S3 Text.
(XLSX)
