# Supplementary material for: Know-how of holding a Bioinformatics competition: Structure, model, overview, and perspectives
Source: PLoS Comput Biol. 2023 Dec 21;19(12):e1011679. doi: 10.1371/journal.pcbi.1011679 (PMC10735175; doi:10.1371/journal.pcbi.1011679)
Supplement: S1 Text — The document presents the competition rules of the LBB 2nd Edition. (DOCX) [file pcbi.1011679.s001.docx]

**Supplementary File 1 - Regulation**

**LEAGUE OF BRAZILIAN BIOINFORMATICS**

**REGULATION**

1. **General Provisions**

1.1 The Brazilian League of Bioinformatics (LBB) is an achievement of the ISCB Regional Student Group Brazil (RSG-Brazil) in partnership with the Brazilian Association of Bioinformatics and Computational Biology (AB3C).

1.2 The planning and execution of LBB and its activities are the responsibility of the Organizing Committee.

1.3 LBB's organizational support structure is located at AB3C's headquarters at Rua do Matão, 1010, Cidade Universitária, São Paulo / SP, CEP: 05508-090.

1.4 LBB is a competition aimed mainly at students from Brazilian universities and the Bioinformatics community.

1.5 The LBB website is https://lbb.ime.usp.br.

1. **Goals**

LBB's main objectives are:

2.1 Stimulate the continuous training of human resources in Bioinformatics through participation in competitions.

2.2 Stimulate and promote the organization of future Bioinformatics competitions, both nationally and internationally.

2.3 Promote the integration of the Bioinformatics community in the country and encourage collaboration between LBB participants.

1. **Participation requirements**

3.1. Teams will be admitted to participate in LBB, under the following conditions:

3.1.1. All participants must be over 18 at the time of registration.

3.1.2. The teams must be composed of 2 or 3 members.

3.1.3. The teams might be composed of members with different educational levels. At least one member has to be enrolled in a higher education institution.

3.1.4. People from any area and academic backgrounds are accepted.

3.1.5. Only 1 (one) member with a complete doctorate per team will be admitted at the registration time (up to two years of degree).

3.1.6. The teams might be composed of members from different institutions.

3.2. The following will not be accepted:

3.2.1. Professionals from research institutes with more than four years of service and employed at the time of registration.

3.2.2. Professionals enrolled in the Bioinformatics area or related areas with more than 4 years of service who are employed at the registration time.

3.2.3 Doctors with more than 2 years of graduation.

3.3. Members of the Organizing Committee and active members of RSG-Brazil are prohibited from participating in LBB.

3.4 Former members of the Organizing Committee and/or RSG-Brazil will be prohibited from participating in the competition under the following circumstances:

3.4.1 Less than 1 year has passed between their official departure from RSG-Brazil and the final registration date of LBB 2021.

3.4.2 Less than 5 years have passed between their official departure from the Organizing Committee of LBB and the final registration date of LBB 2021.

3.5 At least one member must be affiliated with a Brazilian higher education institution.

1. **Subscriptions**

4.1 Registration will be done individually by each team member.

4.2 The group leader must present proof of enrollment in a Brazilian higher education institution.

4.3 It will be necessary to register the team name and a team identification password, as well as the name of all other team members.

4.4 Each member may only register for one team in the LBB. In case of duplication, the member will be disqualified from participating in this year's competition. In case of incorrect registration, the member must submit the correct registration report to the organization.

4.5 The registration form will be available on the LBB website.

4.6 Team registrations will be accepted from 8am (Brasília time) on April 1st, 2021 until 11:59pm (Brasília time) on May 31st, 2021.

4.7 The LBB will not be responsible for registrations not received due to technical problems or network congestion, therefore, it is recommended to submit registrations in advance.

4.8 Only one form per team will be accepted. In the case of duplicate registration, only the last form will be considered.

1. **Duties of the Organizing Committee and LBB Coordinators**

5.1 LBB is composed by the organizing committee, operational coordinators, and partner teachers.

5.1.1 Organization Committee

5.1.1.1 Responsible for organizing the LBB and appointing the board to prepare and correct the questions and challenges.

5.1.1.2 At least one member of the organizing committee will be nominated by the president of RSG-Brazil.

5.1.2 Operational coordinators

5.1.2.1 They will be appointed by the organizing committee, as demanded by it.

5.1.3 Partner professors or postdocs

5.1.3.1 They will be required to elaborate questions for the first and second phases,

according to the needs evaluated by the Organizing Committee.

5.1.3.2 They will be responsible for evaluating the projects developed in the third phase of LBB.

5.1.3.3 They will be appointed by the Organizing Committee.

5.1.3.4 It is a prerequisite to have a PhD for at least two years.

1. **Evidence structure and correction criteria**

6.1 The LBB will be carried out in 3 phases:

6.1.1 First phase

6.1.1.1 The first phase will take place on July 4th, 2021 at 1:00 PM (Brasília time), with a qualifying and elimination character.

6.1.1.2 The exam will last for 5 hours and 3 minutes.

6.1.1.3 The first phase will consist of 60 multiple-choice questions equally divided into three areas: Biology, Computer Science, and Bioinformatics.

6.1.1.4 Each question will be worth 1 point in the first phase score.

6.1.1.5 The ranking of the first phase will be classified according to:

6.1.1.5.1 Teams with equal or more than 50% correct answers in each of the three areas (classified according to the team's total score).

6.1.1.5.2 Teams that did not reach 50% in each area (classified according to the team's total score).

6.1.1.5.3 The submission time of the exam will be used for tie-breaking.

6.1.1.6 If more than 30 teams fit into item 6.1.1.5.1, only teams with scores equal to or higher than 50% correct in each of the three areas (minimum of 10 points in Biology, 10 points in Computer Science, and 10 points in Bioinformatics) will be selected for the second phase. Otherwise, the first 30 teams will be selected for the second phase.

6.1.1.7 The answer form must be submitted only once. In case of multiple submissions by the same group, only the last submission will be considered.

6.1.1.8 LBB will not be responsible for answers not received due to technical problems or network congestion, so it is recommended to submit the exam in advance. 6.1.1.9 The official answer key and results of the first phase will be released the day after the first phase.

6.1.1.10 Resources will be accepted through a form that the organizing committee will make available on the website until the day after the first phase. If all requirements are not met, the resource will be automatically invalidated. Resources will be accepted up to 24 hours after the release of the first phase resource form. Resources sent after this deadline will not be accepted.

6.1.1.11 All resources will be evaluated by the organizing committee within five business days and may lead to the annulment of the question when applicable. The official ranking result will be released within one week after the first phase.

6.1.2 Second phase

6.1.2.1 The second phase will take place over 2 (two) days, September 11th (Saturday) and 12th (Sunday), 2021, with a qualifying and eliminatory character. The test will be made available at 0h (Brasília time) on the 11th and will be available for resolution until 23h59 (Brasília time) on the 12th.

6.1.2.2 The second phase will consist of 5 computational biology challenges.

6.1.2.3 The computational biology challenges will be automatically corrected, and the specific score for each challenge will be specified in the statement. The expected answers to the challenges will be exact or approximate, depending on the challenge.

6.1.2.4 Only the output of each team's solution will be analyzed.

6.1.2.5 The value of each challenge will be made available in the statement of the question.

6.1.2.6 The organization will correct any errors identified in the test correction system during the first 10 hours. Errors identified after this period will be evaluated during the appeal period and may lead to the invalidation of the question. All modifications made during this period will be informed by email to all candidates.

6.1.2.7 The official answers and the score of each team will be available up to 3 days after the end of the test.

6.1.2.8 Appeals will be accepted by filling out the form that the organizing committee will make available on the website up to three days after the end of the second phase. If all requirements are not met, the appeal will be automatically invalidated. Appeals will be accepted within 24 hours after the release of the appeal form.

6.1.2.9 All appeals will be evaluated by the organizing committee within five days after the end of the appeals period and may lead to the invalidation of the question when applicable.

6.1.2.10 In case of inconsistencies in the statements or eventual problems identified by the organizing committee that are not resolved during the test review (as seen in item 6.1.2.6), the questions will be invalidated.

6.1.2.11 Only the top 3 teams with the highest scores will be accepted in the third phase of the LBB. The official result will be available up to one week after the review of appeals.

6.1.2.12 In case of a tie, the following tiebreaker criteria will be used, in this order:

6.1.2.12.1 the lowest number of questions that received a score of zero in the second phase

6.1.2.12.2 the ranking of the first phase.

6.1.2.13 In case of the impossibility of breaking the tie, all teams will move on to the third phase.

6.1.2.14 The LBB is not responsible for answers not received due to possible technical problems or network congestion, so it is recommended to submit the tests in advance.

6.1.3. Third phase

6.1.3.1 The third phase will consist of the development of a project outlined by the Organizing Committee, and the final results of the projects must be presented by the teams on a date and time to be determined by the organizing committee.

6.1.3.2 The third phase will be held online, and the official date will be defined at least 60 days in advance.

6.1.3.3 LBB will not be responsible for technical problems or network congestion during the third phase, so the submission of presentation files should be done within the deadline set by the Organizing Committee.

6.1.3.4 The project presentations must be made in the form of 15-minute seminars, explaining the scientific question and methodologies used, as well as the results obtained.

6.1.3.5 In addition to the oral presentation, a written project of up to five pages will be required and evaluated by the evaluation committee (excluding references).

6.1.3.6 The evaluation criteria will observe:

6.1.3.6.1 The existence of a well-delimited scientific question.

6.1.3.6.2 Adequate choice of methodologies to answer that question.

6.1.3.6.3 Adequate exploration and interpretation of the results obtained.

6.1.3.6.4 Clarity and creativity in the presentation of the project.

6.1.3.7 The judging panel will be responsible for evaluating the participating groups and indicating a winning team.

6.1.3.8 The winner will be announced within 48 hours after the presentations.

6.2 The dates of all phases will be defined in the official calendar but may be subject to change due to unforeseen circumstances. The calendar with all the dates will be announced on the LBB website and social media.

6.3 The results of the three phases, as well as the final score of all participating teams, will be disclosed on the official event website.

1. **Place of the tests**

7.1 All tests will be conducted online on a platform to be disclosed by the Organizing Committee on the official LBB website.

7.2 The tests will only be available for the time set in the schedule, and teams must submit their answers through the indicated platform.

7.3 LBB is not responsible for technical failures that occur during the transmission of answers to the judging panel, and it is entirely the responsibility of the candidate to ensure the submission.

1. **Awards**

8.1 The award ceremony will be held within 48 hours after the final.

8.2 The prizes will be announced on the official LBB website and on social media (Facebook and Instagram).

8.3 Certificates for all participants will be made available in digital format within one month after the end of the competition.

1. **Final considerations**

9.1 By registering for LBB, you agree to:

9.1.1 Comply with the code of conduct and the image authorization term, available at: https://lbb.ime.usp.br/regulamento.

9.1.2 Follow the LBB code of conduct, available at: https://lbb.ime.usp.br/regulamento.

9.2 The calendar of appeals and release of results for the three stages may undergo changes. The official dates will be disclosed on our website and social media at least ten days before each stage.

9.3 Any omissions in this regulation will be analyzed and decided in a sovereign and irrevocable manner by the Organizing Committee of the Brazilian Bioinformatics League.
