# Supplementary material for: Know-how of holding a Bioinformatics competition: Structure, model, overview, and perspectives
Source: PLoS Comput Biol. 2023 Dec 21;19(12):e1011679. doi: 10.1371/journal.pcbi.1011679 (PMC10735175; doi:10.1371/journal.pcbi.1011679)
Supplement: S2 Text — The file contains the script used to create groups of participants following defined criteria. (DOCX) [file pcbi.1011679.s002.docx]

**Supplementary File 2 - Script MATCH**

**This supplementary material refers to the Script for participants who do not have teams but are interested in the competition.**

To cater to participants without teams, LBB introduced solutions to aid in this phase, one of which is LBB MATCH (MATCH). MATCH is an automated Python software that matches candidates based on their traits and preferences, with the primary aim of creating harmonious teams.

Utilizing this data, random selections are carried out to enable team formation, with careful consideration for individual preferences. These selection events involve executing the software to distribute participants into groups of three, always in accordance with the regulations and the participants' preferences. For the current edition, four selection dates have been designated. Following each selection round, profiles of suggested team members are sent, and candidates have the option to either accept or decline the proposed team.

The script below first reads in a list of participants and their relevant details (e.g. name, age, skill level, etc.) from a file or input source. It then uses various criteria to group participants together, such as their level of education or skill level. The specific criteria used will depend on the requirements of the competition and the preferences of the organizer. Once the participants have been grouped together, the script outputs a list of the groups and their members in a .json file. The groups can be outputted to a file or printed to the console for easy viewing.

Overall, this script can be a useful tool for organizing participants into groups for competitions, tournaments, or other events. It can save time and effort for organizers who would otherwise have to manually group participants together.

You can put the questions below in google forms, the questions must follow the order below. So when exporting you will have a spreadsheet, it will be the input file of the script.

In our event we created 3 rounds of responses, where they could accept the team or refuse the profile of the indicated members, the non-response within the deadline it was understood that they would not be interested in that team. If you didn't accept it, you would go to the next round of members, only in the last round, where everyone was presented with names and e-mails, that way we would not compromise personal data.

Moreover, the input data should contain 21 columns with the answers retrieved by a form for the questions below. Simple questions should be answered with ‘Yes’ or ‘No’.

1. Timestamp
2. Email Address
3. First and Last name
4. University/Institute/Work
5. City and State
6. Education level
7. Complete education level
8. Graduation field
9. Which area of bioinformatics are you most knowledgeable about?
10. What is your programming level? (0-10)
11. What programming language?
12. Do you have a completed PhD? (Yes/No)
13. Can you obtain a certificate that you are regularly enrolled in an Institution or University? (Yes/No)
14. Do you mind having teammates from other universities? (Yes/No)
15. Do you mind having teammates from other cities and/or states? (Yes/No)
16. Are you aware that if you accept to work with people from other states and universities, Will you have to work over the internet (Skype, Hangout, Discord, Whatsapp)? (Yes/No)
17. Are you willing to teach your area of work to someone else in the Team? (Yes/No)
18. What levels of education would you prefer to have on your team?
19. Is there any particular area of knowledge that you prefer?
20. Would you prefer to work with someone who has programming skills? (Yes/No)
21. Would you prefer people who have knowledge in which areas of bioinformatics?

**How to run?**

python main.py input_data.csv output_data.json

**Files:**

The "constants.py" file must be in the same directory as the "main.py" file.

**Link:** https://github.com/LeagueBrazilianBioinformatics/MATCH

**Scripts:**

========================== main.py ============================

import pandas as pd

import json

import format

import constants as CONTANTS

import sys

def conta_grupo(grupo):

return len(grupo)

def main():

try:

input_file = sys.argv[1]

except IndexError:

print("Must contain a .csv input file")

return

try:

output_file = sys.argv[2]

except IndexError:

output_file = 'output_file.json'

temp = pd.read_csv(input_file)

len_dados = len(temp)

quantidade_de_grupos = len_dados/3

grupos = {}

lista_membros = []

v = CONTANTS.PhD # Completed PhD?

nome = CONTANTS.name # Name

email = CONTANTS.email # 'Email Address'

cursando = CONTANTS.studying # Education level

completo = CONTANTS.complete # Completed Education Level

#criação dos grupos

for i in range(int(quantidade_de_grupos)):

for j in range(3):

lista_membros.append({'Name and Last name':"---------------"})

grupos['grupo{}'.format(i+1)] = lista_membros

lista_membros = []

# applying the first constraint

# there can only be one person with a PhD

j=0

lista = []

lista_index = []

for i in temp:

lista_index.append(i)

for i in range(len_dados):

if temp[v][i] == ‘Yes’:

lista.append(formatar.formata_dict(lista_index,temp,i))

grupos['grupo{}'.format(j+1)] = lista

j+=1

lista = []

temp = temp.drop([i])

lista_index = []

for i in temp:

lista_index.append(i)

j=0

for i in range(len_dados):

try:

if temp[cursando][i] == ‘Graduation’:

if not grupos['grupo{}'.format(j+1)][0][nome] == '---------------':

lista = grupos['grupo{}'.format(j+1)]

lista.append(formatar.formata_dict(lista_index,temp,i))

grupos['grupo{}'.format(j+1)] = lista

j+=1

lista = []

temp = temp.drop([i])

except:

continue

lista_index = []

for i in temp:

lista_index.append(i)

j=0

for i in range(len_dados):

try:

if temp[cursando][i] == ‘Master’:

for h in range(int(quantidade_de_grupos)):

if not grupos['grupo{}'.format(h+1)][0][nome] == '---------------':

lista = grupos['grupo{}'.format(h+1)]

if len(lista) == 3:

j+=1

lista = []

continue

lista.append(formatar.formata_dict(lista_index,temp,i))

grupos['grupo{}'.format(h+1)] = lista

j+=1

lista = []

temp = temp.drop([i])

except:

continue

j=0

lista_index = []

for i in temp:

lista_index.append(i)

for i in range(len_dados):

try:

if temp[cursando][i] == ‘PhD:

for h in range(int(quantidade_de_grupos)):

if not grupos['grupo{}'.format(h+1)][0][nome] == '---------------':

lista = grupos['grupo{}'.format(h+1)]

if len(lista) == 3:

j+=1

lista = []

continue

lista.append(formatar.formata_dict(lista_index,temp,i))

grupos['grupo{}'.format(h+1)] = lista

j+=1

lista = []

temp = temp.drop([i])

except:

continue

j=0

lista_index = []

for i in temp:

lista_index.append(i)

for i in range(len_dados):

try:

if temp[completo][i] == 'Postdoc':

for h in range(int(quantidade_de_grupos)):

if not grupos['grupo{}'.format(h+1)][0][nome] == '---------------':

lista = grupos['grupo{}'.format(h+1)]

if len(lista) == 3:

j+=1

lista = []

continue

lista.append(formatar.formata_dict(lista_index,temp,i))

grupos['grupo{}'.format(h+1)] = lista

j+=1

lista = []

temp = temp.drop([i])

except:

continue

j=0

lista_index = []

for i in temp:

lista_index.append(i)

for i in range(len_dados):

try:

if temp[cursando][i] == 'Working with Bioinformatics':

for h in range(int(quantidade_de_grupos)):

if not grupos['grupo{}'.format(h+1)][0][nome] == '---------------':

lista = grupos['grupo{}'.format(h+1)]

if len(lista) == 3:

j+=1

lista = []

continue

lista.append(formatar.formata_dict(lista_index,temp,i))

grupos['grupo{}'.format(h+1)] = lista

j+=1

lista = []

temp = temp.drop([i])

except:

continue

lista_index = []

for i in temp:

lista_index.append(i)

for i in range(len_dados):

try:

if temp[cursando][i] == '-':

for h in range(int(quantidade_de_grupos)):

if not grupos['grupo{}'.format(h+1)][0][nome] == '---------------':

lista = grupos['grupo{}'.format(h+1)]

if len(lista) == 3:

j+=1

lista = []

continue

lista.append(formatar.formata_dict(lista_index,temp,i))

grupos['grupo{}'.format(h+1)] = lista

j+=1

lista = []

temp = temp.drop([i])

except:

continue

arquivo_dict = {}

lista_iii = []

for i in grupos:

for j in grupos[i]:

d = str(j['Email Address'])

lista_iii.append({"Email":d if d!='nan' else "",

"nome":j['Name and Last name']})

arquivo_dict[i] = lista_iii

lista_iii = []

conta=0

with open(output_file, 'w') as fp:

fp.write('{')

try:

for i in arquivo_dict:

conta+=1

fp.write('"{}":'.format(i))

fp.write(json.dumps(arquivo_dict[i]))

if conta != len(arquivo_dict):

fp.write("\n,")

else:

fp.write("\n")

print("Results in file:>",arquivo_saida)

except:

fp.write('{},\n'.format(json.dumps(j)))

pass

fp.write('}')

main()

================= constants.py ===================

PhD = 'Do you have a completed PhD?'

name = ‘First and Last name'

email = 'Email Address'

studying = 'Education level'

complete = 'Completed education level'

================= format.py ========================

def formata_dict(lista,dados,i):

dict_data={}

for j in range(len(lista)):

dict_data[lista[j]] = dados[lista[j]][i]

return dict_data
