# Supplementary material for: Know-how of holding a Bioinformatics competition: Structure, model, overview, and perspectives
Source: PLoS Comput Biol. 2023 Dec 21;19(12):e1011679. doi: 10.1371/journal.pcbi.1011679 (PMC10735175; doi:10.1371/journal.pcbi.1011679)
Supplement: S3 Text — This file includes the main analyses of the participants’ scores in the first and second phases. (PDF) [file pcbi.1011679.s003.pdf]

Supplementary File 3 - Assessment of questions

In this supplementary material you will find the Characteristic Curve (ICC) results of the competition participants, the Information Curve (IIC) the responses given by the participants in the first phase of the competition.

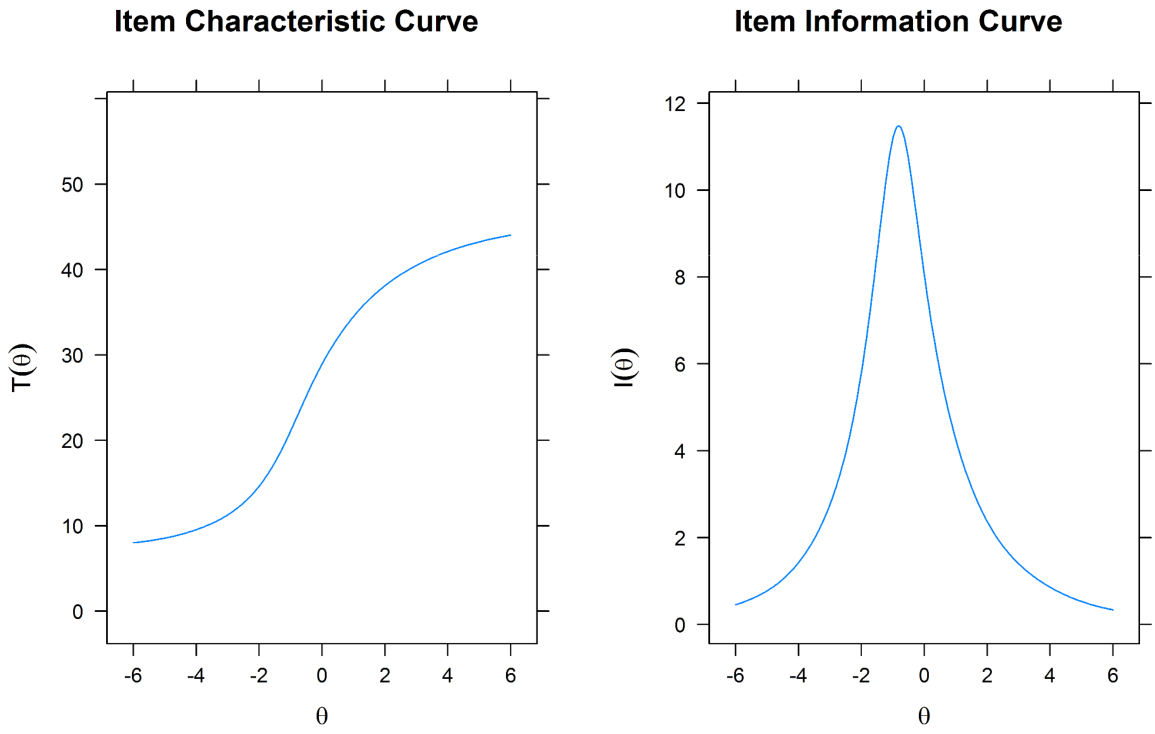

Figure A. (Left) Item Characteristic Curve (ICC) for the responses of the teams participating in the first phase of the LBB. The x-axis represents the team's ability (θ) to achieve a score (T(θ)). Due to the characteristic of the model's curve, we can determine that the first phase test was able to separate participants with low and high ability. (Right) Item Information Curve (IIC) of the 2PL model. The IIC for the whole test shows that the test provides the most information for slightly-higher-than average ability levels (about θ=-1.5), and provides much information about extremely high or low ability levels. The information required for conducting the analysis is available in Table A in S2 Data.

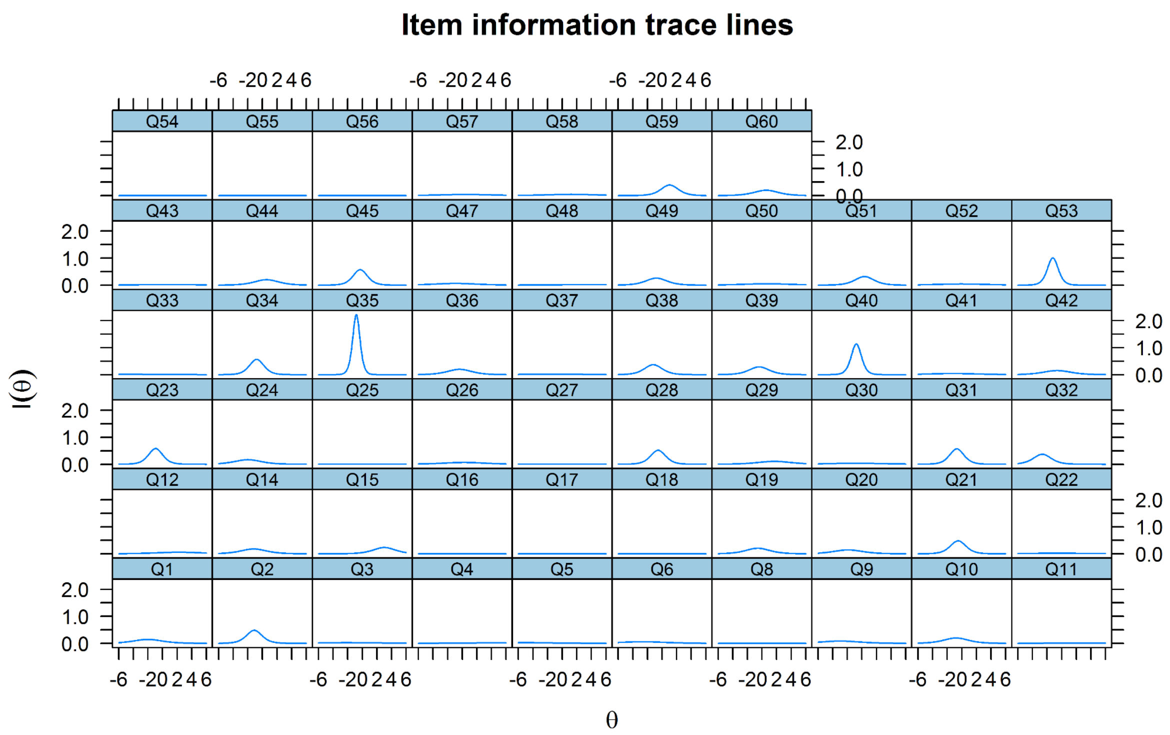

Figure B. Item Information Curve (IIC) for all questions from the first phase of the competition. The x-axis represents the ability θ of each participant and the y-axis the information of a participant with the ability θ aggregate to the model (I(θ)). The greater the information provided by the item, the greater it aggregates to the model. The information required for conducting the analysis is available in Table A in S2 Data.

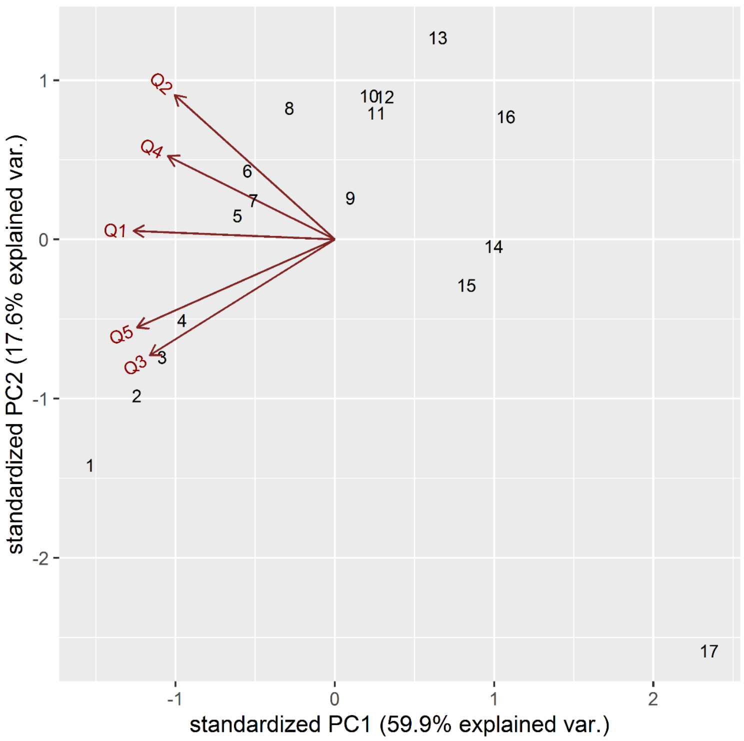

Figure C. PCA biplot from the scores of the 17 groups that obtained a non-zero score in LBB second phase. The labels are in the decreasing order (1st to 17th). Each arrow represents the 5 questions present in the second phase, named as Q_i. The information required for conducting the analysis is available in Table B in S2 Data.
