# Supplementary material for: Know-how of holding a Bioinformatics competition: Structure, model, overview, and perspectives
Source: PLoS Comput Biol. 2023 Dec 21;19(12):e1011679. doi: 10.1371/journal.pcbi.1011679 (PMC10735175; doi:10.1371/journal.pcbi.1011679)
Supplement: S4 Text — The document describes the methods used for the statistical analyses. (DOCX) [file pcbi.1011679.s004.docx]

**Supplementary File 4 - Materials and Methods**

**In this supplementary material we describe how the statistical analyzes present in the article were carried out.**

1. Analyses strategy

1.1 Discrimination of multiple-choice items

Item Response Theory (IRT) is a methodology applied in assessments of different areas to describe the relationship between the level of the latent trait (ability-θ) with the characteristics of the observed items, and the person’s (or group’s) responses to each item (Yang et al., 2014). A reasonable assumption is that each participant who responds to a test item has some amount of associated ability. Thus, each participant can be associated with a numerical value (score), which fits it on the ability scale. This ability score will be indicated by the Greek letter theta (θ). In the first phase test of the LBB all groups were classified according to their ability level with an associated-score ranging from 0 to 60. This score was denoted by T(θ). We could also determine how the probability of a participant with a certain ability θ would provide a correct answer to the item. This probability was denoted by P(θ). In the case of the typical test item, this probability should be small for low-capacity test takers and large for high-capacity test takers (Baker, 2001). The Two Parameter Logistics (2PL) model allows estimating the probability of someone answering an item (of difficulty, b) correctly.

Furthermore, it also allows estimating the discrimination of item (a), which is the ability of an item to distinguish the students that required an ability θ from those who do not have it. The higher the value of "a", the greater the inclination of the Item Characteristic Curve (ICC) and the more discriminant an item is (Zanon et al. , 2016). As a result, most tests used in item response theory consist of multiple-choice items. Therefore, the answers in the first phase were scored dichotomously: the correct answer receives a score of one and an incorrect answer generates a score of zero. From the data transformation, we estimated the 2PL model using the mirt R package (Chalmerset al., 2012) from R 4.1.0..

1.2 Multivariate analysis

Each question in the second phase of LBB was annotated with its respective number according to the order presented in the exam with a letter indicating the order of the sub-question. The 30 teams with scores greater than zero were grouped into three clusters of ten. Teams were named 1 to 30 and sorted by the final score. We labeled the groups into three divisions: (i) Group 1: 1th-10th; Group 2: 11th - 20th and Group 3: 21th-30th. Then, a PCA-biplot was performed using the ggbiplot v0.55 package in R 4.1.0..

1.3 Geospatial Analysis with geobr

We use the geobr v1.6.5 package (Pereira R and Goncalves C, 2023) in R 4.1.0 to generate the map of Brazil with the number of the participants. All data sets in geobr package use the same Geodetic reference system "SIRGAS2000" and CRS(4674), which is the official projection used by IBGE (acronym translated: Brazilian Institute of Geography and Statistics).

2. References

Yang, F. M., & Kao, S. T. (2014). Item response theory for measurement validity. *Shanghai archives of Psychiatry*, *26*(3), 171-177.

Zanon, C., Hutz, C. S., Yoo, H. H., & Hambleton, R. K. (2016). An application of item response theory to psychological test development. *Psicologia: Reflexão e Crítica*, *29*.

Baker, F. B. (2001). *The basics of item response theory*. For full text: http://ericae. net/irt/baker..

Chalmers, R. P. (2012). mirt: A multidimensional item response theory package for the R environment. *Journal of statistical Software*, *48*, 1-29.

Pereira R, Goncalves C (2023). *geobr: Download Official Spatial Data Sets of Brazil*. R package version 1.8.0, https://github.com/ipeaGIT/geobr.
